# Practices, issues and possibilities at the interface between geriatrics and palliative care (InGaP): An exploratory study and knotworking

**DOI:** 10.12688/healthopenres.13534.1

**Published:** 2024-03-21

**Authors:** Erica Borgstrom, Rebekah Schiff, Shaheen A Khan, Esther Hindley, Darmiga Thayabaran, Emily Savage, Nicholas Gough, Richard Holti

**Affiliations:** 1 School of Health, Wellbeing and Social Care, The Open University, Milton Keynes, Buckinghamshire, MK7 6AA, UK; 2 Department of Ageing and Health, Guy's and St Thomas' NHS Foundation Trust, London, UK; 3 Palliative Care Department, Guy's and St Thomas' NHS Foundation Trust, London, UK; 4 Department of Palliative Care, Royal Sussex County Hospital, Univesity Hospital Sussex, Brighton, UK; 5 Faculty of Business and Law, The Open University, Milton Keynes, Buckinghamshire, MK7 6AA, UK

**Keywords:** geriatric medicine, palliative care, end-of-life care, integrated care, older adults, frailty, interdisciplinary working, collaboration

## Abstract

**Introduction:**

With the recognition of the need for palliative care for people with non-malignant conditions, there is an increasing emphasis on interdisciplinary working between geriatric and palliative care teams. This interdisciplinary work has evolved organically; more needs to be known about current working practices. This is of policy and clinical interest as the older patient population continues to grow.

**Methods:**

An exploratory qualitative interview study was undertaken of end-of-life care for older in-patients in a large London NHS Trust. 30 semi-structured qualitative interviews were conducted with staff from palliative care and geriatric medical and nursing teams, two with patients and five with carers. Questions covered: examples and perceptions of collaboration and patient/carer perceptions of clarity as to who was providing care. Interviews were transcribed and thematically analysed focusing on: examples of successful collaboration; areas of tension, duplication or confusion about responsibilities; and suggestions for future practice.

**Results:**

Participants were positive about collaboration. Examples of what works well include: the referral process to the palliative care team; inter-team communication and use of face-to-face handovers; unity between the teams when communicating with patients and families. Areas for potential development include: embedding palliative care within ward multidisciplinary team meetings; continual on-ward education given rotation of staff; and improving collaboration between palliative care, physiotherapy and occupational therapy. It is unclear whether patients’ and carers’ lack of awareness of the different teams has a detrimental effect on their care or needs.

**Conclusions:**

There is evidence of strong collaborative working between the teams; however, this study highlights potential areas for improvement. An exploration of these relationships in other settings is required to determine if the same themes arise with a view to inform national guidelines and policy to improve care towards the end of life.

## Plain Language Summary

There is a need for geriatric and palliative care teams to work together. How they have done this so far has developed over time, but little is documented about how they work together. We conducted interviews at a large London hospital to look at end-of-life care for older persons. 30 staff, who worked either in palliative care or geriatric teams, were interviewed as were two patients and five carers. Interview questions asked about collaboration and how care was provided. Researchers identified themes in the interviews, looking for examples of successful collaboration, areas of tension or confusion, and suggestions for future changes. Interviewees were positive about collaboration, especially when it came to referring to palliative care and communication about specific patients. It is unclear whether patients’ and carers’ lack of awareness of the different teams has a negative effect on them. There is evidence of strong collaborative working between the teams; however, this study highlights potential areas for improvement. More research in this area is needed to inform national guidelines and policy.

## Introduction

With the expansion of palliative care into non-malignant conditions and the recognition that end-of-life care is ‘everyone’s business’ ( National Palliative and End of Life Care Partnership, 2021; Oliver, 2016), there is an increasing emphasis on interdisciplinary working. This change is particularly relevant to geriatric medicine, a specialty experienced in providing healthcare for the growing population of older adults living with frailty, many of whom are towards the end of their lives. How these two specialisms optimise working together to improve outcomes for this population is vital to informing policy and practice as it develops on wards. It is presumed in the medical literature that both specialities share similar goals about patient care and could work well together. For over 20 years, there has been a call for more research on the overlap between geriatric medicine and palliative care ( Albers *et al*., 2016; Goldstein & Morrison, 2005; Seymour *et al*., 2001). 

However, a recent literature review has shown that little is known empirically about how these two specialisms work together in practice, with existing literature focusing mainly on North America or the training of healthcare professionals ( Visser *et al*., 2020). Yet, while both specialisms are perceived as inherently interdisciplinary, published knowledge on actual daily working practices revealing how these two specialisms interact, especially in the UK, is limited. Existing literature has sought to shed light on two related questions – (i) how to improve access to palliative care for older patients in acute hospitals ( Goldsmith *et al*., 2010), and (ii) what kind of collaboration between palliative care specialists and geriatricians is most effective. The exploratory research in this article focuses on question (ii) on the basis that better understanding of this question will improve what is required for answering question (i).

Both specialisms work within acute hospital wards and community settings; with continuous restructures of care, teams may span these boundaries. What is known about the current interface is that boundaries of work between the specialisms are unclear and that communication between professionals and teams is paramount for collaborative working ( Visser *et al*., 2020). Current literature suggests that new models of interdisciplinary working between geriatrics and palliative care are being developed both consciously and as emergent adaptations to current practice ( Bone *et al*., 2016; Goldsmith *et al*., 2010). However, there is as yet only weak evidence of what precisely these collaborative models are and how much they vary across and within different contexts. Most importantly the effectiveness of such models in improving patient care, from both professional and patient perspectives, has not been explored. By studying how these two specialisms operate within one hospital with a focus on working practices, this study provides an insight into how integrated working at the end of life for older patients can be done and what areas can be further explored.

## Methods

### Background to the study site

This project is based on research conducted within one London hospital within one NHS (National Health Service) Trust. The trust is unusual compared to many NHS trusts in that both the geriatric medicine and palliative care teams are quite well-established. The palliative care team is one of the oldest within UK hospitals, and therefore has had a relatively long time to embed itself within the hospital culture. However, this is not without its challenges, and this has changed over the years. The two specialist teams have developed working relationships organically over the years. This study arose out of a request from the Palliative Care team to researchers at The Open University in 2017 to help them understand their working practices.

Each specialist team covers both acute and community settings, with some members of the teams transitioning between settings. Through discussions with the clinical leads of both teams, it was evident that the most apparent overlap in end-of-life care is within the Older Persons Unit (OPU), a three ward area of 84 beds, within one hospital. The project team decided to focus on this setting for the exploratory study to enable an examination of where there is noticeable overlap and collaboration to understand current working practices further. Along with the Open University researchers, Schriff, Khan and Gough helped design the research questions and study protocol. The study received a favourable opinion from the university and HRA ethics boards (HREC/2755/Holti and IRAS 229254 respectively). Participants provided written informed consent to partake in the study and to have their interview recorded, transcribed, and anonymised and for anonymised quotes to be used in research communications. Due to the nature of the study (identifiable location and professional roles), to protect participant confidentiality, raw data cannot be made accessible through any data repository; the participant information sheet informed people that ‘all recordings and verbatim transcriptions will be held as confidential to the OU research team.’ Hence, participants were not consented for their data to be made publicly available; exemplar longer anonymised quotes for data verification are available via request to the corresponding author. As per the data management plan, transcripts will be held until 2028.

The exploratory study consisted of semi-structured qualitative interviews with staff from both specialist teams as well as semi-structured interviews with patients (or those close to them, such as family or informal carers) who had recently been under the care of both teams within the Older Persons Unit. The research team sought to interview staff across roles and level (exact details or a participant table are not provided to protect participant anonymity; it included medical, nursing and allied health professionals) and several patients/carers in receipt of care from both teams. Recruitment, especially of patients and carers, was facilitated by Hindley, Thayabaran and Savage who provided study information, participant information sheets, and booked interview rooms. Recruitment of staff was via email. Recruitment of patients and carers was via printed information provided to them whilst in the Older Persons Unit; they were informed that participation was voluntary, and that participation (or not) would not impact care provided. Hindley, Thayabaran and Savage were neither a clinical lead nor responsible for data collection to minimise power dynamics influencing participation.

### Data collection

Thirty semi-structured qualitative interviews were conducted with staff, two with patients and five with carers either by Borgstrom or Holti, or both (in the case of interviewing clinical leads). Questions covered: recent examples where teams worked together; staff perceptions of collaboration and issues; patient and carer perceptions of clarity as to who was providing care ( Borgstrom & Holti, 2024). For the purposes of this study, and to be in line with policy understandings of end-of-life care ( Department of Health, 2008), we defined end-of-life care as care for any person in the last 12 months of life. All interviews lasted between 15–60 minutes and were conducted within private spaces within the hospital (unless a patient or carer preferred to do a telephone interview), were audio-recorded with consent, and later transcribed verbatim by a third-party company specialising in transcription. Transcripts were checked and anonymised by the interviewer; participants could request a copy of their transcript. All interviews were conducted within a two-month timespan (2017–2018). 

### Data analysis

Borgstrom and Holti analysed the transcripts. Initial analysis involved each interviewer reading interviews they conducted, as well as a small subset of the other interviews, to identify themes to examine further. These themes along with exemplifying quotes were shared and refined to provide a coding structure. All interviews were then thematically analysed by Borgstrom focusing on working practices. Latterly, we applied activity theory to the data to make sense of the working practices (see Discussion). Whilst there were additional themes in the data – such as how palliative or end-of-life care is perceived within the hospital – the focus of this exploratory study is on working practices with the intent to inform collaboration between palliative and geriatric medicine. An executive summary of the thematic analysis was shared with clinical leads and project collaborators to refine the focus of further analysis for publication. Findings were also shared to help inform ongoing clinical partnerships, with this being the primary focus ahead of academic publishing.

## Results

Within the interviews with professionals, we found examples of successful collaboration, areas of tension, duplication or confusion about responsibilities and suggestions for future practice. We have also dedicated a section to the patient and carer perspectives that span across these other themes as they point to a different way of understanding how collaboration works, is made visible (or not), and utilised by patients/carers.

### Successful collaboration

Participants were overwhelmingly positive about collaboration between the teams. Examples of what participants thought works well were: the referral process to the palliative care team; inter-team communication and use of face-to-face handovers; and unity between the teams when communicating with patients and families. The palliative care team were thought of as responsive and visible. For example,

‘…you always get a response from the palliative care team…they’re very visible when they’re on the ward and they usually come and say hello and…we’ll talk to them. So I think it’s quite an open relationship and…I think I get along well with them and we have open discussions about patients…’ (Participant 07).

The referral process to palliative care was viewed as enabling staff on the Older Persons Unit to ‘escalate appropriately’ in the context of end of life (Participant 05). Participants in geriatric medicine also commented on the expertise of palliative medicine and that they valued the ‘second opinion’ that their colleagues could provide. This was deemed particularly helpful for junior doctors to build their confidence in identifying the end of life and for ‘presenting a unified front’ to provide ‘the same message to the family members’ (Participant 03).

Staff in both professional fields talked about the usefulness of their conversations together that focused on patient care. From both perspectives it was viewed as a kind of ‘teaching opportunity’ (Participant 12), especially when done face-to-face and covering what to do and why for a patient as part of handovers. In these discussions, they were also able to flexibly and mutually establish roles – e.g. who would speak to the family. Moreover, our respondents noted that each team appeared to have regular internal updates about patients which meant that even if they spoke to someone else on the team, they were knowledgeable enough to understand the situation and provide guidance. It is important to note that most of what was reported as working well was around communication practices between and within the teams and focused on furthering patient care priorities.

### Areas of tension

Staff participants were able to be reflective about where there were areas of tensions in the relationship between the two specialities. These included: the variability of referrals received by the palliative care team; delays in specialist opinions and patient discharge; and some professionals feeling unsure about their relationship with and to palliative care.

At the time of the study, the referral process to palliative care involved sending a form to the team which they triaged and frequently followed up with a phone call. Whilst staff on the Older Persons Unit generally viewed this as straightforward, once they understood how to use the form, staff within the palliative care teams discussed the variability in amount and type of referrals they received from each of the three wards on the Older Persons Unit. For example, one participant appreciated the knowledge of some of the geriatrician clinicians that did not frequently refer to palliative care in the hospital but noted that this could ‘muddy the waters’ (Participant 01) when liaising with community palliative care and reporting to commissioners. When referrals were made, some staff on the Older Persons Unit expressed a concern about needing to wait for the palliative care team before they could act further. For instance, ‘…sometimes you are waiting on a specialist’s opinion that would delay medications being prescribed or medications being given’ (Participant 05). So, whilst staff expressed acknowledgement that the referral process was easy to use, the use of it was variable and what to do whilst it was being processed could also lead to tenson.

The palliative care team were also perceived at times to ‘delay discharge’ of a patient once involved on the Older Persons Unit, which both teams appreciated could be frustrating. From the palliative care team’s perspective, this was often caused by a misunderstanding of what palliative care do and the need to establish the basis for the referral. This was articulated by one palliative care clinician as follows (in the context of receiving a referral to support discharging patients home):

      ‘…[it’s] managing expectations between the team…not wanting to delay things, which we certainly don’t, but also wanting to be safe by discharging people home…we might highlight new things [because of our holistic approach] that need to be addressed…’ (Participant 12).

Some palliative care staff also felt frustration when their advice following a referral, even for discharge, was not acted upon. They recognised that their authority in implementing their own advice could be limited:

      ‘….it is quite infuriating if we come back in the next day and they [the recommendations] haven’t been actioned. Be we don’t have control because we are an advisory service’ (Participant 01).

This led some staff to wonder about the reason for inaction. This included concerns about time allotted to care tasks on the ward, difficulties in reading notes in a timely manner, and levels of knowledge of ward staff about palliative care practices. It was beyond the scope of the interview-based study to know what led to these examples; however, some interviewees did note that they preferred if communication between staff did not rely solely on notes in patient records, especially as it could be hours between opportunities that busy staff have to log in and view notes. Others noted that if palliative care nurses left the note for ward-based nurses, that sometimes it was not clear who has the authority to action that recommendation, or if the palliative nurse would do it themselves. Overall, regardless of reasons, these perceived inactions or delayed acting on advice appeared to impact how successful some people felt in partnership working.

### Roles and responsibilities

In all interviews, participants talked about different roles and responsibilities. People felt confident in their role and understanding of their responsibility on a day-to-day basis. As the above section indicated, there could be at times questions about what others’ roles were, and this was due to what some perceived as ‘siloed’ working (Participant 02) when it came to the specialists, especially as palliative care did not attend the ward multidisciplinary team meetings. Some staff on the Older Persons Unit expressed confusion about what their role may be once a patient is referred to palliative care. For example, allied health professionals had sometimes felt like they were no longer needed or thought of as part of the patient’s care team once the person was identified to be near the end of life. However, the staff themselves saw that there could be merit in their continued involvement.

In some patient cases, staff told us that there was confusion about whether or not staff who visit the ward would know if the patient is being seen by the palliative care team. This depended on the ability to see this readily in the electronic notes and make sense of how this would be impacting the patient’s care management. In other examples, staff noted that there could be confusion around whether a patient could continue to receive physiotherapy or occupational therapy, especially after discharge, because they ‘were being looked after by the palliative care team’ (Participant 06) and what the new ceiling of treatment may be. To help resolve such queries, the teams would discuss the case in multidisciplinary team meetings and calls to the palliative care team; notably, the allied health professionals did not speak directly to palliative care unless they happened to see them on the ward.

Staff on the wards viewed the palliative care team as having the ability to play a crucial role in discharges, especially for ‘fast track discharges’ (where a patient was thought to be dying within the next six weeks). This was because palliative care staff were deemed to know how to fill in the forms required to access funding for short term additional support at home or in a care home. The palliative care team were also considered to have useful knowledge of and links to community services. If a discharge was to a care home though, it was acknowledged that this required a multidisciplinary approach because staff were required ‘to identify what their care needs actually are…from cognitive impairment to personal care to mobility to function…’ (Participant 20).

Staff on the ward also found it useful to involve the palliative care team when meeting with patients’ families and communicating about the end of life. Staff noted that the palliative care team often had more time for such communication. They also found that family liked to talk to them, for example, to ‘have smaller conversations about pain relief’ (Participant 11). Yet, when it came to patient and carer perspectives, few distinguished between geriatric medicine staff, ward staff, and the palliative care team. However, there was one case where we found the patients’ family member knew what palliative care represented and were able to use this to leverage different types of care for their family member, such as being able to advocate for comfort care and support services upon discharge. 

### Suggestions for future development

Areas of concern and for future development included: embedding palliative care within multidisciplinary team meetings within the ward; the need for continual on-ward education given rotation of junior medical staff; improving collaboration between palliative care, physiotherapy and occupational therapy; patients’ and carers’ lack of awareness of the different teams and whether this has a detrimental effect on their care. Suggestions for future development came from participants in a wide range of roles and teams rather than being driven by one group or another.

Embedding palliative care into ward multidisciplinary team meetings was suggested partly to enable more immediate collaboration. For example:

      ‘in an ideal world you would want the palliative team to be part of the [multidisciplinary team] meetings, if that can be done, fantastic. Because we get sufficient numbers of palliative patients nowadays that it would be worth their while attending the [meetings]. Like we have dieticians in there, probably we have less diet problems than we actually have palliative care problems on the ward.’ (Participant 03)

It was noted that other specialists, such as occupational and physical therapists were part of these meetings, and being present could help provide more collaboration between these roles as well. Being part of ward meetings was also perceived to provide opportunities for ongoing, informal education and could facilitate better understanding about palliative care services available in the community.

It was also suggested that palliative care could be more present within the wards by providing more formal education and raising awareness about the team. This was especially wanted by new and/or rotating staff who may not have been in post when previous training was delivered. Training of ward staff was viewed as ‘upskilling [them] to feel more comfortable and confident and talking openly about death and dying and kind of, so they know what to expect as well’ (Participant 12) in addition to some more specific training on symptom management. The training was not suggested to diminish the role of specialist palliative care, but rather to upskill colleagues to an extent that referrals to the team could therefore be focused on the key contributions of specialist care.

From the interviews with patients and carers, it was apparent that they did not necessarily recognise the staff as being part of different teams, although where they did know this, this knowledge could be used to leverage access to specialist services. For example, one carer understood what palliative care was and therefore proactively asked for their involvement. Within the interviews, staff participants were not clear whether patients and their carers knew or did not know the distinction between the staff groups or whether these perceptions of role mattered overall. Interviewed staff expressed a preference for when family meetings could be done with both teams, and generally making it clear to patients and families that staff had relevant expertise for the situation at hand or knew how to access such expertise.

## Discussion

The research literature suggests that there is limited understanding between the two specialisms (palliative care and geriatrics) in what they can offer each other ( Bosch *et al*., 2009) with interactions often taking place in an informal and ad-hoc way ( Albers *et al*., 2016). There are a variety of views within each specialty, as well as between them, regarding the most appropriate role for collaboration in end-of-life care. Some geriatricians are reported as not seeing end-of-life care as part of their job, and hence keen for palliative care specialists to step in, whilst others see a palliative approach to end-of-life care as entirely within their repertoire of expertise and experience ( Goodwin *et al*., 2014). Conversely, some palliative care specialists are reported as staking claim to a distinctive bundle of medical and psychological expertise, whilst others are keen to educate, up-skill and support other specialists to provide the basic elements of palliative and end-of-life care ( Goodwin *et al*., 2014). Our findings show that a collaborative approach is often mutually desired, and at times is felt to be present, particularly fostered by specific individuals and behaviours. However, there are also indications that collaboration also needs to be underpinned by processes and infrastructures that support it to work well.

One way of making sense of this is through activity theory and the concept of knot-working. Activity theory provides a framework for analysing work, acknowledging both the micro-level processes and macro-level structures that inform work and how work is done in activity systems ( Engeström, 2000). In the data presented above, using activity theory we can identify that there are particular ways of working – for example, how to send and receive referrals to palliative care – that operate as ‘standard scripts’ that people know how to do, but that this knowing is both learned and evolving, as circumstance or contingencies emerge that in some way challenge the script, as for example when an initially straightforward discharge of a dying person home becomes more complex because issues emerge concerning difficult family dynamics. Variations emerge between how individuals interpret and accomplish a script, whilst the main outcome (achieving palliative care input for patients on the Older Persons Unit) remains valid. The suggestions from participants about more palliative care education can be interpreted as a desire to inform people’s understanding of the ‘standard scripts’ around palliative care provisions within the Older Persons Unit, including the kinds of contingencies and fine-grained judgements that may be involved.

More specifically, Engestroem’s concept of knotworking can elucidate ‘the process of tying and untying various threads of activity and knowledge from across the MDT [multidisciplinary team] in order to accomplish specific objectives over time’ ( Mnaymneh *et al*., 2021). The central knot can be the patient. In this context, knotworking generally involves people who are loosely connected to come together at different points (and then disconnect again) to, for example, manage patient care, sometimes making complex and finely-balanced judgements, often involving the relatively indeterminate views and priorities of patients and their carers, alongside more determinate or technical medical issues. The length of interaction between staff can be relatively brief, even only a few minutes long ( Hurlock-Chorostecki *et al*., 2015). Knotworking has been shown in other studies to be a more effective way of understanding collaboration compared to concepts like networking or traditional team building, as the types of teams that form around in-patient care can be fluid, rather than always involve the exact same members of staff ( Bleakley, 2014), spontaneous and short-lived ( Hurlock-Chorostecki *et al*., 2015). The challenge for management then is to foster practices that can facilitate knotworking through building team rapport, sharing of knowledge, a sense of clear roles and responsibilities but also the ability to be both a leader and a follower ( Varpio & Teunissen, 2021), maintain a focus on shared objectives, and deliberate readily across a range of medical, social, psychological and family system issues. Importantly, and supported by our findings, knotworking may appear like invisible work ( Engeström, 2018) and patients may not recognise the wide team that supports them unless present in a joint meeting.

Within our data, we observe knotworking as occurring around several key points of activity. These include referral processes from Older Persons Unit to palliative care, information provision from palliative care to staff on the Older Persons Unit to inform decision-making about specific patient care, discharge planning, and joint patient/family meetings attended by both Old Persons Unit and palliative care staff. Where people described examples of successful collaboration, this can be interpreted as moments of successful knotworking. For example, successful knotworking is illustrated by the ability to have palliative care provide a ‘second opinion’, where different expertise comes together via individual practitioners. Where participants described areas of tension between the teams, this can be interpreted as times when the ‘tying of the knots’ was unsatisfactory for the participant. For instance, they may have felt that different individuals from parts of the wider team did not engage with them much (i.e. allied professionals and palliative care not collaborating explicitly). Or there could have been a lack of clarity around who does what once they attempt to ‘work around the knot’/patient, and a lack of appreciation of different ways of working (e.g. holistic assessment) to achieve the same objective of patient care and discharge. Appreciating these moments as knotworking – inherently requiring the coming together around specific threads of activity – can help identify the inter-professional nature of work and practical solutions for future practice.

The suggestions for future directions indicated above were provided in presentations and short reports to both palliative care and the Older Persons Unit clinical leads shortly after data collection and initial analyses were complete. This has helped inform ongoing practices, although some changes were heavily impacted by the re-organisation that has occurred since COVID. One area that was suggested was embedding palliative care into the multidisciplinary meetings; at the time, this was deemed impractical due to the wide range of MDTs across the hospital that the palliative care team fed into and clash of timings. The suggestion about training highlighted the importance of not viewing training as a one-off event (see also Miller *et al*., 2010; Mlambo *et al*., 2021; Zeiger, 2005) and opened up ways of thinking about how training could be delivered to provide knowledge and skills as well as relationship building.

Others have looked at inter-professional collaboration through the lens of service ecosystems, outlining more of the structural aspects that influence collaboration. When using this perspective to study palliative care, researchers have found that there are several factors that impact palliative care services when collaborating with others, including coordination of work, resource integration, and the ability to communicate value ( Sudbury-Riley & Hunter-Jones, 2021). They suggest that value co-creation is important and can be supported institutionally. Our findings on knotworking can contribute to understanding how this co-creation is created and maintained in daily working practices around patient care and hospital processes. 

Strengths of this study are that it included perspectives of a wide range of staff in both the Older Persons Unit (geriatrics team) and palliative care team, thereby not only focusing on clinical leads’ perspectives. This was helpful for capturing examples and suggestions for changes that may have otherwise been missed or underrepresented. 

Study limitations include only focusing on one hospital and using only interviews for data collection. Whilst findings about successful collaborations and tensions may be applicable to other sites, we cannot generalise about all collaborations between geriatrics and palliative care in acute hospitals. The findings of this research are intended as a first stage in addressing this gap – focusing on one acute hospital setting - and will pave the way for defining further research. Additionally, observational data of staff interactions could provide further insight into how knotworking functions, the issues it needs to address, and where it could be improved further.

## Conclusions

In many countries, like the UK, the number of older persons who are facing the end of life is growing and many of them will be in hospital at some point during their last year of life. To support older patients towards and at the end of life, geriatrics and palliative care teams are likely to continue to work closely together, both within the hospital and beyond via discharge arrangements. Successful collaboration is a result of successful knotworking – knowing when and how to come together around different threads of activity to support a common objective of patient care.

Importantly, knotworking is not about sustained collaboration between specific members of staff but the ability to work together at key moments. In our study examples of this included being responsive to each other, providing expertise and education, and holding joint meetings with families. Yet more research is needed to understand further the conditions, both structural and inter-personal, that give rise to the ability to tie effective knots around staff engaged in addressing an issue, recognising the complex range of social and medical issues involved in end-of-life care for older people. Since there is increasing pressure the management of limited healthcare resources and interest in patient outcomes towards and at the end of life, examining interdisciplinary collaboration through the lens of knotworking provides a means to understand working practices between teams when caring for older persons.

## Data Availability

Due to the nature of the study (identifiable location and professional roles), to protect participant confidentiality, raw data cannot be made accessible through any data repository; the participant information sheet approved by the ethics committees informed people that ‘all recordings and verbatim transcriptions will be held as confidential to the OU research team.’ Hence, participants were not consented for their data to be made publicly available; exemplar longer anonymised quotes for data verification are available via request to the corresponding author. Reason for the request and volume of data requested (e.g. number of quotes) to be provided upon contacting the corresponding author. Legitimate requests include: reviewing the manuscript (by a reviewer linked to the journal and/or an academic institution); requests for data to enable secondary analysis are not permissible. Access to quotes will be time-limited and via encrypted services; the requester must agree not to use the quotes for any other purpose, and not to seek to de-anonymise participants. As per the data management plan, transcripts will be held until 2028. Requests for data after this time will not be possible. The interview schedule is available in the ORDO (Open Research Data Online Repository) ( Borgstrom & Holti, 2024). https://doi.org/10.21954/ou.rd.25050446.v1 Data are available under the terms of the Creative Commons Attribution 4.0 International license (CC-BY 4.0)
