# Supplementary material for: Practices, issues and possibilities at the interface between geriatrics and palliative care (InGaP): A case study exemplifying knotworking
Source: Health Open Res. 2025 Oct 8;6:12. Originally published 2024 Mar 21. [Version 2] doi: 10.12688/healthopenres.13534.2 (PMC12680095; doi:10.12688/healthopenres.13534.2)
Supplement: Peer review history

# Reviewer report: Reviewer response for version 2

**DOI:** 10.21956/healthopenres.15038.r29676

**Published:** 2025-11-25

**Authors:** Marie-Louise Möllerberg

**Affiliations:** 1 Malmö University, Malmö, Sweden

Thank you for the opportunity to review this manuscript. This is an important and timely study given the growing need for integrated approaches in geriatrics and palliative care. The current article, “Practices, issues and possibilities at the interface between geriatrics and palliative care (InGaP): A case study exemplifying knotworking”, presents a qualitative case study exploring interdisciplinary collaboration between geriatric and palliative care teams in a large London NHS Trust. Thirty staff interviews, two patient interviews, and five carer interviews were conducted to examine working practices using activity theory and the concept of knotworking.

 The study identifies examples of successful collaboration (e.g., referral processes, communication, joint family meetings) and areas for improvement (e.g., unclear roles, delays in discharge, lack of integration in MDT meetings). Recommendations include embedding palliative care in MDT meetings, ongoing education for rotating staff, and improved collaboration with allied health professionals. This work is highly relevant for informing policy and practice aimed at improving end-of-life care for older adults.

 1.    Is the work clearly and accurately presented and does it cite the current literature?

 Partly. The manuscript is well-written and cites relevant literature, including recent sources. However, the abstract lacks an explicit research question and does not mention the guiding theoretical frameworks. Some references in the discussion are dated and could be supplemented with more recent evidence.

 2.    Is the study design appropriate and is the work technically sound?

 Partly. The qualitative case study approach is appropriate, but the methods section lacks detail. There is insufficient information on sampling strategy, interviewer characteristics, and how data saturation was assessed. The rationale for using only interviews should be explained.

 3.    Are sufficient details of methods and analysis provided to allow replication by others?

 Partly. Thematic analysis is described, but details on coding procedures, use of software, and saturation are missing.

 4.    Statistical analysis and interpretation:

 Not applicable. This is a qualitative study.

 5.    Are all source data underlying the results available to ensure full reproducibility?

 No source data required. Ethical constraints prevent sharing raw data, but anonymized quotes are available upon request. This is acceptable but should be stated more clearly in the methods.

 6.    Are the conclusions drawn adequately supported by the results?

 Yes. Conclusions are consistent with findings and discussion. However, the first paragraph of the discussion could be more concise, and transferability should be emphasized rather than generalizability.

 Key Points That Must Be Addressed

 Abstract:

 Add explicit research question and aim.

 Mention activity theory and knotworking.

 Introduction:

 State objectives clearly at the end.

 Methods:

 Detail sampling strategy and justify.

 Describe interviewer characteristics and prior relationships.

 Explain why only interviews were used and acknowledge limitations.

 Add information on coding process and saturation.

 Results:

 Include thematic map and more participant quotes.

 Discussion:

 Start with a concise summary of key findings.

 Emphasize transferability rather than generalizability.

 Update references and link findings to recent policy changes.

 Conclusion:

 Highlight practical implications for clinical practice and policy.

Is the work clearly and accurately presented and does it cite the current literature?

Partly

If applicable, is the statistical analysis and its interpretation appropriate?

Not applicable

Are all the source data underlying the results available to ensure full reproducibility?

No source data required

Is the study design appropriate and is the work technically sound?

Partly

Are the conclusions drawn adequately supported by the results?

Yes

Are sufficient details of methods and analysis provided to allow replication by others?

Partly

Reviewer Expertise:

Palliative care

I confirm that I have read this submission and believe that I have an appropriate level of expertise to confirm that it is of an acceptable scientific standard, however I have significant reservations, as outlined above.

# Reviewer report: Reviewer response for version 2

**DOI:** 10.21956/healthopenres.15038.r29587

**Published:** 2025-11-19

**Authors:** Sarah Combes

**Affiliations:** 1 University of Surrey, Guildford, England, UK

Thank you for the opportunity to re-review the paper. The amendments have, for me, made the work much clearer and more impactful. Thank you.

Is the work clearly and accurately presented and does it cite the current literature?

Partly

If applicable, is the statistical analysis and its interpretation appropriate?

Not applicable

Are all the source data underlying the results available to ensure full reproducibility?

No

Is the study design appropriate and is the work technically sound?

Yes

Are the conclusions drawn adequately supported by the results?

Partly

Are sufficient details of methods and analysis provided to allow replication by others?

Yes

Reviewer Expertise:

Palliative and end of life care mixed methods research; Older people; Older people living with complex needs; Advance care planning; Workforce development

I confirm that I have read this submission and believe that I have an appropriate level of expertise to confirm that it is of an acceptable scientific standard.

# Reviewer report: Reviewer response for version 2

**DOI:** 10.21956/healthopenres.15038.r29586

**Published:** 2025-11-08

**Authors:** Sophie Pask

**Affiliations:** 1 University of Hull, Hull, UK

Thank you for the opportunity to review the revised manuscript. I have some further comments in relation to the amendments and recommend some minor changes. These relate mostly to the case study approach and its description:

 1. The authors could elaborate on whether their qualitative case study is descriptive or explanatory. This could then be articulated within the abstract and throughout the manuscript.

 2. In the introduction, the sentence 'The case study in this article speaks to question (ii) on the basis that better understanding of this question will improve what is required for answering question (i). In doing so, our focus is on understanding interprofessional working practices by analysing a case study using activity theory ( Engeström, 2000) rather than patient outcomes.' could come at the end to lead into the methods. The last paragraph describing the benefit of a case study approach and knotworking feels more suited to methods.

 3. The authors briefly acknowlegde the limitations of only using interviews, this could be elaborated on further in terms of their case study approach. They may also benefit from a brief justification in their methods of why only qualitiative interviews were used as case studies often use multiple methods. 

 4. In the limitations, I would recommend reframing to transferability of findings rather than generalising.

 I feel that the authors have addressed other concerns/comments.

 Thank you again for the opportunity to review this pertinent and interesting piece of work.

Is the work clearly and accurately presented and does it cite the current literature?

Partly

If applicable, is the statistical analysis and its interpretation appropriate?

Not applicable

Are all the source data underlying the results available to ensure full reproducibility?

No

Is the study design appropriate and is the work technically sound?

Yes

Are the conclusions drawn adequately supported by the results?

Partly

Are sufficient details of methods and analysis provided to allow replication by others?

Yes

Reviewer Expertise:

Palliative and end-of-life care research

I confirm that I have read this submission and believe that I have an appropriate level of expertise to confirm that it is of an acceptable scientific standard, however I have significant reservations, as outlined above.

# Reviewer report: Reviewer response for version 2

**DOI:** 10.21956/healthopenres.15038.r29591

**Published:** 2025-11-05

**Authors:** Nikolaos Vlachopoulos

**Affiliations:** 1 251 Hellenic Air Force General Hospital, Athens, Greece

General feedback:

 In total, the manuscript is scientifically robust and technically sound. This is a well-written, coherent, and timely qualitative case study examining interdisciplinary collaboration between geriatric and palliative care teams in an NHS hospital. It makes a valuable contribution to the underexplored empirical interface between these specialisms.

 The abstract is structured properly providing all necessary information. However, there are some points to consider:

- Abstract lacks an **explicit research question**; authors might add a short paragraph highlighting the purpose of the study:
  - ***Aim:*** *This qualitative case study explored interdisciplinary collaboration in end-of-life care for older inpatients within a large London NHS Trust, drawing on activity theory and the concept of knotworking to understand how integration is enacted in daily practice.*
- No mention is made of the **guiding frameworks ( *activity theory* or *knotworking*),** which weakens the methodological transparency from the outset.
- The abstract should explicitly state that this is a **qualitative study conducted at a single case site**

Keywords are also provided. Relevant references and literature. Neither ethical issues nor competing interest issues are observed. Plagiarism is not suspected.

Feedback on the introduction/background/rationale/theoretical framework:

 Nice work. An effective introduction and rationale, relevant background, and references on the topic of the survey. All necessary information is provided, but, for the sake of the coherence of the text, it would be beneficial if t **he research objectives are were explicitly formulated at the end of the last paragraph as questions or aims *(e.g., “This study aims to examine how collaboration occurs between geriatric and palliative care teams to deliver end-of-life care for older patients in an acute hospital setting. Using activity theory and the concept of knotworking, we seek to understand how professionals negotiate roles, responsibilities, and communication processes in practice”)***

Feedback on the design, methods and statistics:

 Research design and survey were direct with proper analysis, but not well organized. Authors may consider **subdividing into *Design*, *Setting and Participants*, *Data Collection*, *Analysis*, and *Ethical Considerations*.**

 In general, a great effort has been made to conduct a rich and comprehensive statistical analysis. However, there are a few considerations for improvement:

- I recommend that researchers utilize the following evaluation checklist, which is designed for critiquing qualitative studies, to improve their article: **COREQ (COnsolidated criteria for REporting Qualitative research) Checklist.**
- The manuscript lacks information about the interviewers' characteristics. Describe the interviewers' experience, training, qualifications, and relevant personal characteristics (e.g., gender, cultural background) that might have influenced the research process.
- State whether the interviewers had any prior contact or relationship with the participants before the study. If so, describe the nature of the relationship.
- The sampling strategy is not accurately described. Clarify the sampling strategy. Was it purposive, convenience, or a combination of both? If a combination, please explain how both methods were used and justify this choice.
- Provide more details about the data collection process, including: How and why the interview method was chosen., How rapport was established with participants., How participant feedback was incorporated into the process.
- There is no detail regarding how saturation was evaluated. The manuscript does not provide an adequate justification for the inclusion of only two patients and five carers in the study sample.

Feedback on the main body of the manuscript:

 The section provides rich, well-selected quotations and strong thematic organization. Nevertheless, τhere are a few points I would like to comment on. At first, I would rather see a **thematic map **depicting the nuances and complexities of each challenge. Additionally, it is recommended that more quotations from different participants be included in each section of the results. This will enhance the rigor of your study.

 In general, the Discussion successfully situates findings within existing literature and clearly conveys implications for practice. However, the first paragraph of the Discussion seems a bit chaotic. I would prefer a **concise paragraph in which only the main elements and results of the study will be discussed. This paragraph should concisely and explicitly answer the question: *What are the key takeaways from the research? *** Subsequently, the detailed discussion of the results can continue in the manner in which you have presented it.

Feedback on the conclusions/summary:

 Relevant and interesting conclusions and interpretations from the study. It is always difficult to build a message and evaluate an indicator from a single study in this field. Overall, the topic is of interest for the scientific community, especially in preparation of geriatric population is growing.

Is the work clearly and accurately presented and does it cite the current literature?

Yes

If applicable, is the statistical analysis and its interpretation appropriate?

Not applicable

Are all the source data underlying the results available to ensure full reproducibility?

No source data required

Is the study design appropriate and is the work technically sound?

Partly

Are the conclusions drawn adequately supported by the results?

Yes

Are sufficient details of methods and analysis provided to allow replication by others?

Partly

Reviewer Expertise:

Primary care, Internal Medicine, Cardiology

I confirm that I have read this submission and believe that I have an appropriate level of expertise to confirm that it is of an acceptable scientific standard, however I have significant reservations, as outlined above.

# Reviewer report: Reviewer response for version 1

**DOI:** 10.21956/healthopenres.14621.r28851

**Published:** 2024-11-12

**Authors:** Sophie Pask

**Affiliations:** 1 University of Hull, Hull, UK

Thank you for the opportunity to read this interesting and relevant paper exploring the interdisciplinary working practices of geriatric and palliative care teams. This is an important topic as we aim for more integrated and coordinated care. This paper provides an insight into how this interface is being managed in one particular hospital site, drawing on both the positives and potential challenges that may need to be addressed. I have some reflections that I hope will help to refine this paper:

1. The paper focuses on a specific Trust that you describe as ‘unusual’ compared to many other NHS Trusts. In addition, there is some ambiguity around the role of the limited number of patient and carer interviews in the development of themes. The paper may be better framed as a case study or service evaluation to understand examples of good practice and/or areas for improvement.
2. The data are interesting and provide a useful reflection on the interface between these teams. However, there have been substantial changes in integrated working since the period of data collection (with the introduction of Integrated Care Boards), as well as the impact of the pandemic. This raises questions around the relevance and application of findings from this data – perhaps you can reflect more on whether these data still apply and how? Although, I note that you refer to presentations/short reports to clinical leads after data collection, and acknowledge the impact of COVID. 
3. The reflection on lenses to review inter-professional collaboration and knot working might be better placed earlier on in the methods section of the paper, and how it guided your thinking and the analysis.
4. I would also welcome further clarification on whether quotes presented are from professionals, patients or carers. Also, the reason behind the limited number of patients and carers recruited to this study (e.g. challenges to recruitment), and acknowledging this as a potential limitation.
5. Although you highlight relevant literature, my concern is noting that there is limited understanding with the use of more dated references in the discussion. I would recommend updating some of the references used. You may find the following reference helpful also: Evans CJ, Ison L, Ellis-Smith C, Nicholson C, Costa A, Oluyase AO, Namisango E, Bone AE, Brighton LJ, Yi D, Combes S, Bajwah S, Gao W, Harding R, Ong P, Higginson IJ, Maddocks M. Service Delivery Models to Maximize Quality of Life for Older People at the End of Life: A Rapid Review. Milbank Q. 2019 Mar;97(1):113-175. doi: 10.1111/1468-0009.12373. 

Is the work clearly and accurately presented and does it cite the current literature?

Partly

If applicable, is the statistical analysis and its interpretation appropriate?

Not applicable

Are all the source data underlying the results available to ensure full reproducibility?

No

Is the study design appropriate and is the work technically sound?

Yes

Are the conclusions drawn adequately supported by the results?

Partly

Are sufficient details of methods and analysis provided to allow replication by others?

Yes

Reviewer Expertise:

Palliative and end-of-life care research

I confirm that I have read this submission and believe that I have an appropriate level of expertise to confirm that it is of an acceptable scientific standard, however I have significant reservations, as outlined above.

## References

[1] : Service Delivery Models to Maximize Quality of Life for Older People at the End of Life: A Rapid Review. *Milbank Q* .2019;97(1) : 10.1111/1468-0009.12373 113-175 10.1111/1468-0009.12373 30883956 PMC6422603

# Reviewer report: Reviewer response for version 1

**DOI:** 10.21956/healthopenres.14621.r28667

**Published:** 2024-10-18

**Authors:** Sarah Combes

**Affiliations:** 1 University of Surrey, Guildford, England, UK

Thank you for this opportunity to read your interesting paper regarding the interface between palliative and older persons' care. This is an extremely important topic as we strive towards person-centred and integrated care, and it has been interesting to see how this interface is being enacted in one London acute hospital, and the attendant challenges and opportunities they have faced. 

 While I enjoyed reading the paper I do have some concerns. My key concerns are:

1. The paper reports the views of staff, with minimal patient carer views, of one London acute hospital, in one Trust, which the authors say themselves is an "unusual" trust. It is an interesting paper, but I wonder if, in its current form, it is more of a service evaluation assessing how well this specific service was performing in 2017-2018, rather than research that is generating new knowledge that can be applied more generally. 
2. The data is from 2017-2018. We have had significant policy changes since then, let alone a global pandemic. Is this data from one hospital in London still relevant? Does the service work in the same way? Where is the links to the community and care closer to home, where most older people would rather be? Where are the links/relevance to Integrated Care Boards/Services etc? 
3. The authors suggest little work has been conduced in this area yet do not reference several key recent references Ref (1) Common elements of service delivery models that optimise quality of life and health service use among older people with advanced progressive conditions: a tertiary systematic review doi:10.1136/bmjopen-2020-048417.
4. It is sometimes not clear to see who the participants are. I believe it may be that all participants are staff, with patient/carer voices noted in the second half of one paragraph on page 6, which is repeated almost word for word in the first half of one para on page 7. It would be helpful if the authors could make the "participant" role clearer for the reader.
5.  The analysis is explained elegantly in the Discussion, rather than the methods section. It would help the reader to describe activity theory and knotwork earlier.

As previously stated, the paper is interesting, and it is elegantly written in areas. I do wonder though, could this be reimagined as a paper focused on activity theory and knotwork with the interviews/research used as a case example to demonstrate the theory? The Discussion and Conclusion already reads like this may be the case.

Is the work clearly and accurately presented and does it cite the current literature?

Partly

If applicable, is the statistical analysis and its interpretation appropriate?

Not applicable

Are all the source data underlying the results available to ensure full reproducibility?

No

Is the study design appropriate and is the work technically sound?

Yes

Are the conclusions drawn adequately supported by the results?

Partly

Are sufficient details of methods and analysis provided to allow replication by others?

Yes

Reviewer Expertise:

Palliative and end of life care mixed methods research; Older people; Older people living with complex needs; Advance care planning; Workforce development

I confirm that I have read this submission and believe that I have an appropriate level of expertise to confirm that it is of an acceptable scientific standard, however I have significant reservations, as outlined above.

## References

[1] : Service Delivery Models to Maximize Quality of Life for Older People at the End of Life: A Rapid Review. *Milbank Q* .2019;97(1) : 10.1111/1468-0009.12373 113-175 10.1111/1468-0009.12373 30883956 PMC6422603

[2] : Common elements of service delivery models that optimise quality of life and health service use among older people with advanced progressive conditions: a tertiary systematic review. *BMJ Open* .2021;11(12) : 10.1136/bmjopen-2020-048417 e048417 10.1136/bmjopen-2020-048417 34853100 PMC8638152
